# Investigation of the Multi-Target Mechanism of Guanxin-Shutong Capsule in Cerebrovascular Diseases: A Systems Pharmacology and Experimental Assessment

**DOI:** 10.3389/fphar.2021.650770

**Published:** 2021-05-13

**Authors:** Juanli Zhang, Jiaxin Zhao, Yang Ma, Wenjun Wang, Shaojie Huang, Chao Guo, Kai Wang, Xiaomei Zhang, Wei Zhang, Aidong Wen, Ming Shi, Yi Ding

**Affiliations:** ^1^ Department of Pharmacy, Xijing Hospital, Fourth Military Medical University, Xi’an, China; ^2^ Department of Neurology, Xijing Hospital, Fourth Military Medical University, Xi’an, China; ^3^ Basic Medical School, Yunnan University of Traditional Chinese Medicine, Kunming, China

**Keywords:** guanxin-shutong capsule, multi-target mechanism, cerebrovascular diseases, systems pharmacology, experimental assessment

## Abstract

Guanxin-Shutong capsule (GXSTC), a combination of Mongolian medicines and traditional herbs, has been clinically proven to be effective in treating cerebrovascular diseases (CBVDs). However, the underlying pharmacological mechanisms of GXSTC in CBVDs remain largely unknown. In this study, a combination of systems pharmacology and experimental assessment approach was used to investigate the bioactive components, core targets, and possible mechanisms of GXSTC in the treatment of CBVDs. A total of 15 main components within GXSTC were identified using high-performance liquid chromatography coupled with diode array detector (HPLC-DAD) and a literature research. Fifty-five common genes were obtained by matching 252 potential genes of GXSTC with 462 CBVD-related genes. Seven core components in GXSTC and 12 core genes of GXSTC on CBVDs were further determined using the protein-protein interaction (PPI) and component-target-pathway (C-T-P) network analysis. Gene Ontology (GO) and Kyoto Encyclopedia of Genes and Genomes (KEGG) pathway enrichment analysis results predicted that the molecular mechanisms of GXSTC on CBVDs were mainly associated with the regulation of the vascular endothelial function, inflammatory response, and neuronal apoptosis. Molecular docking results suggested that almost all of core component-targets have an excellent binding activity (affinity < −5 kcal/mol). More importantly, in middle cerebral artery occlusion (MCAO) -injured rats, GXSTC significantly improved the neurological function, reduced the infarct volume, and decreased the percentage of impaired neurons in a dose-dependent manner. Western blotting results indicated that GXSTC markedly upregulated the expression of vascular endothelial growth factor A (VEGFA) and endothelial nitric oxide synthase (eNOS), while downregulating the expression of cyclooxygenase-2 (COX-2) and transcription factor AP-1 (c-Jun) in MCAO-injured rats. These findings confirmed our prediction that GXSTC exerts a multi-target synergetic mechanism in CBVDs by maintaining vascular endothelial function, inhibiting neuronal apoptosis and inflammatory processes. The results of this study may provide a theoretical basis for GXSTC research and the clinical application of GXSTC in CBVDs.

## Introduction

Cerebrovascular diseases, characterized by stroke and other forms of neurological dysfunction and degeneration, are among the most common causes of morbidity and mortality worldwide, seriously affecting human health and life (Collaborators., G.B.D.N., 2019). Until now, intravenous thrombolysis with recombinant tissue plasminogen activator (rt-PA) has been the main treatment option for acute CBVDs. However, owing to the limited therapeutic window (<4.5 h after symptom onset) and the hemorrhagic complications induced by rt-PA, only a small number of stroke patients can benefit from this treatment (Hankey, 2017). In addition, most neuroprotective drugs have been demonstrated to be effective in animal experimental studies but their effectiveness could not be demonstrated in clinical trials (Zhou et al., 2018). Therefore, there is a pressing need to develop safer and more effective drugs for CBVDs.

Traditional Chinese medicine (TCM) has been used for thousands of years to prevent and treat CBVDs. Guanxin-Shutong capsule, a combination of Mongolian medicines and traditional herbs, has been commonly used to treat angina pectoris and coronary heart diseases in China (Li et al., 2019); it consists of *Choerospondias axillaris* (Roxb.) B.L.Burtt and A.W.Hill [*Anacardiaceae; Choerospondiatis fructus*], *Salvia miltiorrhiza Bunge* [*Lamiaceae; Radix Salviae miltiorrhizae*], *Syzigium aromaticum* (L.) Merr. and L.M.Perry [Myrtaceae*; Caryophylli flos*], *Dryobalanops aromatica* C.F.Gaertn [Dipterocarpaceae; *Bomeolum*], and *Bambusa textilis* McClure [Poaceae*; Concretio silicea bambusae*] (Rivera et al., 2014) at a weight ratio of 16:8:2:1:1 by weight. The major active components of GXSTC include phenolic acids, tanshinones, saponins, and others (Gao et al., 2017). In recent years, several clinical trials have further demonstrated that GXSTC has a significant clinical effect in treating ischemic stroke, and could markedly improve the nerve function of stroke patients (Wu, 2015; Shao, 2018). However, the effective components and potential mechanisms of action of GXSTC against CBVDs remain unclear.

Systems pharmacology is an effective tool for drug-target analysis with the advantages of a low cost, short cycle, and providing more comprehensive information. Moreover, it has gradually promoted the paradigm shift from the “one-target, one-drug” mode to a “network-targets, multiple-components” approach in drug discovery (Kibble et al., 2015; Zhang et al., 2019). Coincidentally, the holistic strategy of systems pharmacology is in accordance with the combinatorial therapeutic strategies of TCM. Therefore, it has been widely used to investigate the complex pharmacological mechanisms of TCM (Yuan et al., 2017). Several studies have successfully revealed the mystery of TCM formulae for treating CBVDs based on systems pharmacology, such as Danhong injections (Wei et al., 2016), Shuxuening injections (Cui et al., 2020), and Naoxintong capsules (Xu et al., 2016).

**FIGURE 1:**
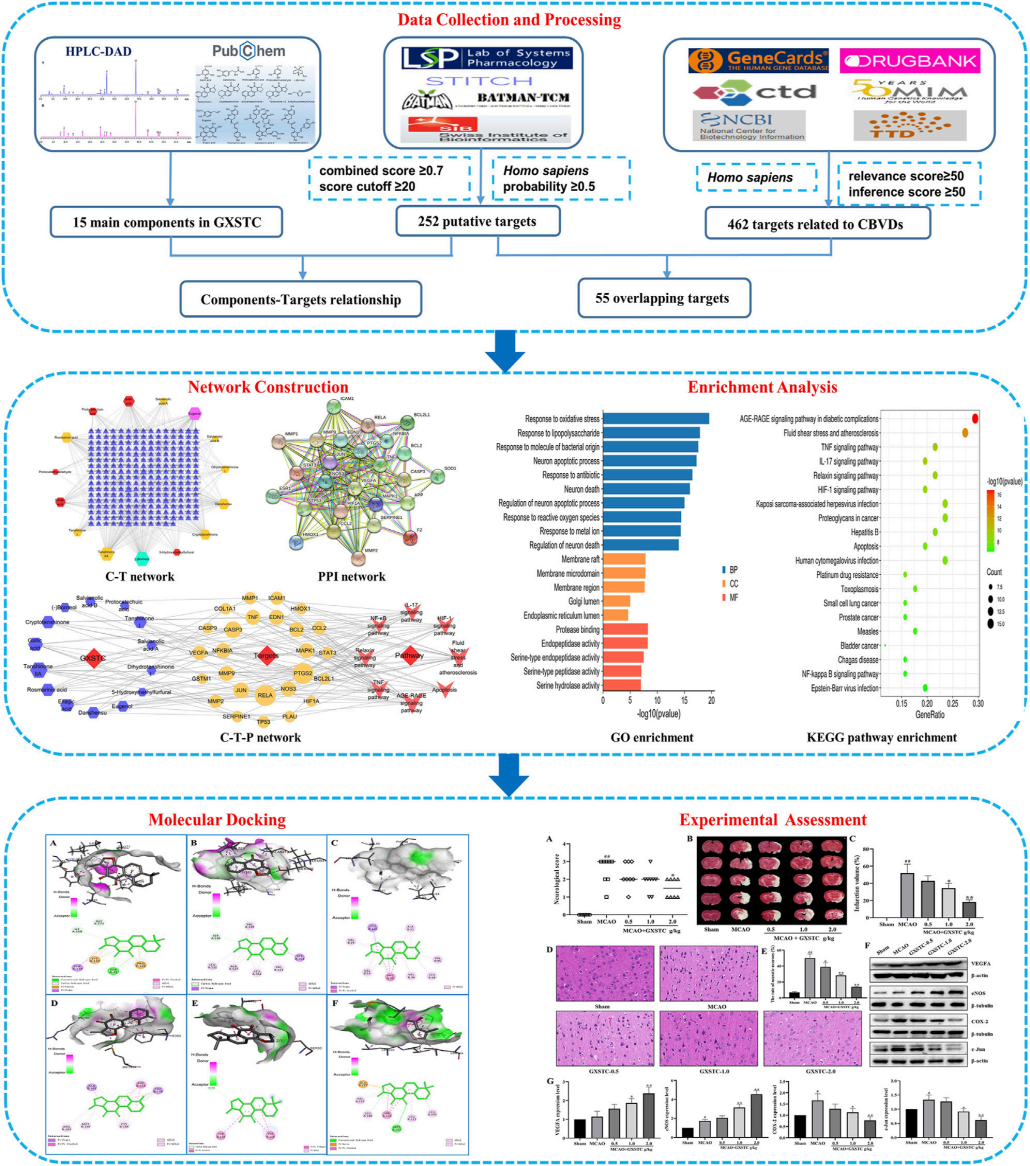
Workflow of systems pharmacology analysis and experimental assessment.

In this study, the mechanisms of action of GXSTC on CBVDs were investigated by combining systems pharmacology and experimental assessment. The detailed workflow is shown in Figure 1. First, the main components of GXSTC were identified by using HPLC-DAD to determine standard samples. The potential targets of these components and therapeutic targets related to CBVDs were collected from multiple databases. Subsequently, 55 common targets were identified by matching the potential targets of GXSTC with the CBVD-related targets. On this basis, network construction and enrichment analysis were performed to systematically investigate the underlying interactions between bioactive components, key targets, and pathways. The interactions between the core components and the corresponding core targets were evaluated using molecular docking. Finally, an animal model of ischemic stroke was used to investigate the molecular mechanism of GXSTC in CBVDs.

## Materials and Methods

### Chemicals and Materials

GXSTC was supplied by Buchang Pharmaceuticals (Xianyang, China). The reference standards of gallic acid, ellagic acid, tanshinone IIA, cryptotanshinone, protocatechuic acid, and eugenol were obtained from the National Institute for the Control of Pharmaceutical and Biological Products (Beijing, China). The reference standards of danshensu, 5-hydroxymethylfurfural, protocatechualdehyde, rosmarinic acid, salvianolic acid A, salvianolic acid B, tanshinone I, and dihydrotanshinone I were purchased from Baoji Chenguang Biotechnology Co. Ltd. Deionized water was purified by a Milli-Q water purification system (Millipore, MA, United States). Acetonitrile and methanol (HPLC-grade) were purchased from Fisher Scientific (Fair Lawn, NJ, United States). 2,3,5-triphenyltetrazolium chloride (TTC, purity > 98.0%) was obtained from Sigma-Aldrich (St. Louis, MO). A bicinchoninic acid (BCA) protein assay kit and a sodium dodecyl sulfate polyacrylamide gel electrophoresis (SDS-PAGE) kit were bought from Dingguo Changsheng Biotechnology (Beijing, China). The running buffer (pH: 8.4–8.6), transfer buffer (pH: 8.4), and Tris-buffered saline (TBS) were purchased from Servicebio (Wuhan, China). Anti-VEGFA (ab46154) were bought from Abcam (Cambridge, MA, United States). Anti-β-actin (13E5) and anti-c-Jun (60A8) rabbit mAbs were bought from Cell Signaling Technology (CST, Beverly, MA, United States of America). Anti-eNOS (GB11086) and anti-COX2 (GB11077-2) rabbit polyclonal antibodies were obtained from Servicebio. HRP-conjugated AffiniPure goat anti-rabbit IgG (H + L, SA00001-2) was purchased from ProteinTech Group (Chicago, IL, United States). Anti-β-tubulin and other reagents were supplied by other Chinese suppliers.

### Determination of the Main Components of GXSTC

The GXSTC powder (0.5 g), and a mixture of 14 reference standards were dissolved in 50 ml methanol, respectively. After sonication for 30 min, the samples were filtered through 0.22 μm syringe filter, and 20 μL filtrate was injected into the HPLC-DAD system for analysis. HPLC analysis of GXSTC was performed by LC-20AT HPLC system (Shimadzu, Kyoto, Japan) with an Agilent TC-C_18_ column (4.6 × 250 mm, 5 μm). The mobile phase was composed of solvent A (acetonitrile) and solvent B (water containing 0.05% phosphoric acid) and the gradient procedure was set as follows: 0–15 min, 5–20% A; 15–18 min, 20–34% A; 18–30 min, 34–40% A; 30–35 min, 40–50% A; 35–40 min, 50–65% A; 40–60 min, 65–75% A; the UV detection wavelength was set at 274 nm.

### Target Prediction of GXSTC Components

First, we obtained the molecular structures and canonical SMILES codes of all the components from PubChem (https://pubchem.ncbi.nlm.nih.gov/) (Kim et al., 2019). Next, the targets related to these components were collected by using TCMSP (http://tcmspw.com/tcmsp.php, ver. 2.3) (Ru et al., 2014), BATMAN-TCM (http://bionet.ncpsb.org/batman-tcm/, ver. 2016) (Liu et al., 2016), STITCH (http://stitch.embl.de/, ver. 5.18) (Kuhn et al., 2008), and Swiss Target Prediction (http://www.swisstargetprediction.ch/, ver. 2019) (Daina et al., 2019) databases with the species limited to “*Homo sapiens*”. In these databases, the predicted interactions between components and targets were attributed to “score cutoff,” “combined score,” or “probability” with a higher score indicating a stronger interaction. In the present study, only the targets with a score cutoff ≥20 in BATMAN-TCM, a combined score ≥0.7 in STITCH, and a probability ≥0.5 in Swiss Target Prediction databases were selected.

### Candidate Target Collection of CBVDs

The targets related to CBVDs were acquired from six databases, which aimed to shed light on the relationship between targets and diseases from different perspectives. “cerebrovascular diseases” or “cerebrovascular” was used as a keyword to search the OMIM (http://omim.org/) (Amberger and Hamosh, 2017), GeneCards (https://www.genecards.org/) (Stelzer et al., 2016), NCBI Gene (https://www.ncbi.nlm.nih.gov/gene/) (Brown et al., 2015), TTD (http://bidd.nus.edu.sg/group/cjttd/) (Wang et al., 2020), and Drugbank (https://www.drugbank.ca) (Wishart et al., 2006) databases. “Cerebrovascular disorders” was used as a keyword to search the CTD (http://ctdbase.org/) (Davis et al., 2019). To ensure the reliability of target prediction, we only chose the targets with a relevance score ≥50 in GeneCards and an inference score ≥50 in the CTD database, respectively. Finally, all the protein names were standardized into official gene symbols and IDs using UniProtKB (https://www.uniprot.org/) (Bateman et al., 2019) database by confining the species to “*Homo sapiens*.” After removing the duplicate targets, the common targets related to CBVDs and the candidate components were identified as the candidate targets.

### C-T and PPI Network Construction

To understand the complicated interactions between components and their corresponding targets, the component-target (C-T) network was established by using Cytoscape (https://cytoscape.org/, ver. 3.7.2) (Franz et al., 2016). In addition, the candidate targets were uploaded to the STRING database (https://string-db.org/, ver. 11.0) (Szklarczyk et al., 2019) to obtain the PPI data by confining the species to “*Homo sapiens*” and a high confidence score ≥0.7. The PPI data were obtained, and the PPI network was constructed through Cytoscape. Moreover, the degree values of nodes were calculated in the network, and the targets with a degree above their median were selected as key targets of the PPI network.

### GO Functional and KEGG Pathway Enrichment Analysis

To investigate the potential molecular mechanisms of GXSTC on CBVDs, candidate targets were analyzed for GO functional and KEGG pathway enrichment analysis based on the “cluster profiler” and “org.Hs.eg.db packages” of the R project (https://www.r-project.org/, ver. 3.6.2) (Mair et al., 2015). The enrichment *p*-value was calculated, and a *p* < 0.05 was considered to be statistically significant. Finally, a component-target-pathway (C-T-P) network was constructed to investigate the reciprocity of the components, targets and pathways by using Cytoscape.

### Molecular Docking Studies

To evaluate the reliability of the predicted interactions between the core components and their corresponding targets, molecular docking was performed by using AutoDock Vina 1.1.2 (Trott and Olson, 2010). First, the 3D structures of core components (ligands) were downloaded from PubChem and stored in a PDB file format, and then converted to MOL2 files by using ChemBioDraw Ultra 14.0. The crystal structures of the core targets were obtained from the RCSB Protein Data Bank (https://www.rcsb.org/) (Rose et al., 2017). Then, the components and target proteins for docking were prepared by using PyMOL 1.7.2.1, and they were saved as PDBQT files using AutoDockTools 1.5.6. Finally, a docking simulation was performed under AutoDock program, and the lower affinity score for the component-target was selected for further analysis. The action modes of the core components with their corresponding targets were analyzed and visualized by using Discovery Studio Visualizer.

### Experimental Assessment

#### Experimental Design

Adult male Sprague-Dawley rats with a body weight of 250–280 g were supplied by the Experimental Animal Center of the Fourth Military Medical University. Rats were housed under controlled conditions with a 12 h light/dark cycle at 25 ± 2°C and 50 ± 10% humidity. The experimental protocols were approved by the Ethics Committee for Animal Experimentation of the Fourth Military Medical University and were performed according to the National Institutes of Health Guide for the Care and Use of Laboratory Animals. Rats were randomly divided into five groups (*n* = 8 in each group): sham, middle cerebral artery occlusion, and MCAO + GXSTC pretreatment (0.5, 1.0, and 2.0 g/kg per day). These doses were chosen based on the dosage of GXSTC in clinical practice (0.3 g/capsule, 3 capsules once, 3 times a day) (Li et al., 2019) and previous studies (0.5–2.0 g/kg in rats) (Zhuo et al., 2012; Zhu et al., 2020). Rats in the sham and MCAO groups were pretreated with 0.9% saline solution, and rats in the GXSTC groups were pretreated with GXSTC (0.5, 1.0, and 2.0 g/kg per day, respectively) via intragastric administration for 7 days. The rats were then anesthetized, and a MCAO was established as previously described (Guo et al., 2017). After 2.0 h of MCAO, the filament was withdrawn to allow reperfusion for 24 h. The sham group underwent the same surgical procedure except that the filament was not inserted into the internal carotid artery.

#### Neurological Deficit Score

After reperfusion, the neurological deficits were scored using a 5-point scale scoring system, as described previously (Longa et al., 1989). The detailed scoring criteria were as follows: 0, no deficit; 1, failure to fully extend the left forepaw; 2, circling to the left; 3, falling to the left; and 4, no spontaneous walking with a depressed level of consciousness.

#### Infarct Volume Assessment

After reperfusion, the rats were deeply anesthetized and decapitated. The brains were rapidly removed and stored on ice for 15 min. The brains were cut into 2 mm coronal slices, stained with 2% TTC solution for 30 min, and then fixed with 4% paraformaldehyde solution at 4°C overnight. After staining, the slices were arranged in groups and photographed, and the infarct volume of each slice was measured by using Image Pro Plus 6.0. To exclude the confounding effect of brain edema, the corrected infarct volumes were calculated through the following formula:

$$\text{Measured infarct volume}\times \left(1-\frac{\text{ipsilateral hemisphere volume}–\text{contralateral hemisphere volume}}{\text{contralateral hemisphere}}\right)$$

Finally, the infarct volume (%) in each group was expressed as the percentage of contralateral hemispheric volume.

#### Histological Observation

To evaluate the histological damage, rats were deeply euthanized and sacrificed at 24 h after reperfusion. Subsequently, the brains were quickly removed and immediately immersed in 4% paraformaldehyde. After fixation for 24 h, the brain tissues were dehydrated, embedded in paraffin and cut into 5 μm coronal slides. Finally, the brain slides were stained with H&E, and the histopathological changes were observed by using light microscopy at a ×400 magnification.

#### Western Blot Analysis

After 24 h of reperfusion, the right brain tissues were pooled and homogenized via sonication in RIPA lysis buffer (Beyotime, China) containing protease and phosphatase inhibitors (Vazyme Biotech, Nanjing, China). The total protein concentration in each sample was measured by a BCA protein assay kit according to the manufacturer’s instructions. Equal amounts of protein samples (30–60 μg per sample) were separated by using 8–10% SDS-PAGE gels and transferred to PVDF membranes. The membranes were blocked with 5% nonfat milk in TBS containing 0.1% TWEEN (TBST) at room temperature for 1 h, and then incubated with the following primary antibodies: anti-VEGFA (1:1,000), e-NOS (1:500), c-Jun (1:1,000), COX-2 (1:1,000), *β*-actin (1:1,000), and *β*-tubulin (1:1,000) at 4°C overnight. Next, the membranes were rinsed in TBST for 10 min three times, and then incubated with HRP-labeled secondary antibody (goat anti-rabbit IgG, 1:10,000) for 1 h at room temperature. Finally, the blots were visualized by using an enhanced chemiluminescence method (Diyibio, Shanghai, China) and were analyzed using the ImageJ software (version 1.46r, National Institutes of Health, United States).

#### Statistical Analysis

Statistical analysis was performed using GraphPad Prism 8.0.1 for Windows. All data, except for the neurologic score values, were expressed as mean ± standard deviation. Statistical differences between the various groups were analyzed using one-way analysis of variance (ANOVA). Statistical significance was set at *p* < 0.05, and a significant statistical significance was set at *p* < 0.01.

## Results

### HPLC Profile of GXSTC

**FIGURE 2:**
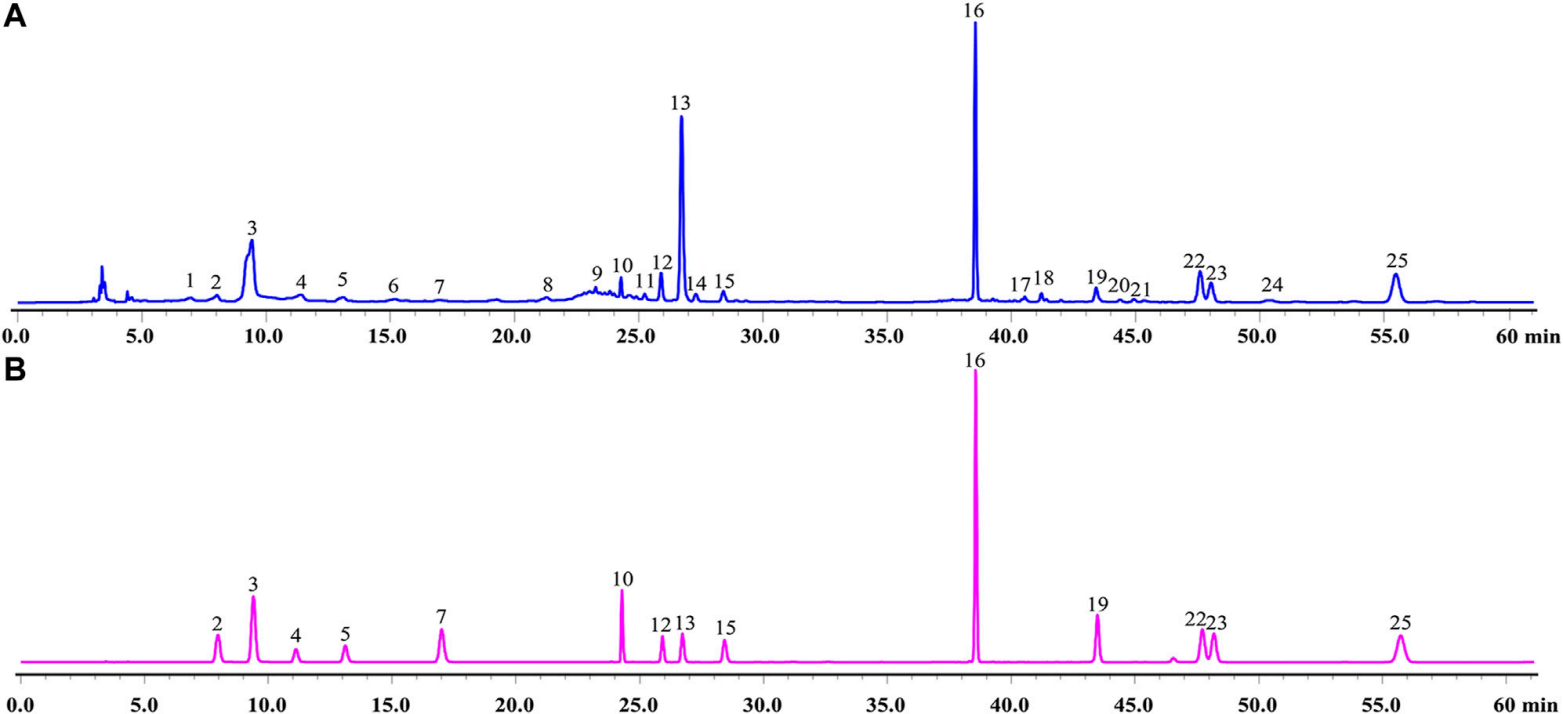
HPLC chromatograms of GXSTC **(A)** and mixed standard sample **(B)** at 274 nm. Mixed standard sample was consisted of gallic acid (2), 5-Hydroxymethylfurfural (3), danshensu (4), protocatechuic acid (5), protocatechualdehyde (7), ellagic acid (10), rosmarinic acid (12), salvianolic acid B (13), salvianolic acid A (15), eugenol (16), dihydrotanshinone I (19), cryptotanshinone (22), tanshinone I (23), tanshinone IIA (25).

A total of 14 main components in GXSTC were unambiguously identified by comparing the retention time with those of standard samples (Figure 2). Based on the TCMSP databases and on literature research, five components were primarily derived from *Choerospondiatis fructus*, eight components were primarily produced by *Radix Salviae miltiorrhizae*, and two components were derived from *Salviae miltiorrhizae*. However, only tanshinone IIA, salvianolic acid B, and borneol were used as marker components for the quality control of GXSTC in the Chinese Pharmacopeia. Among them, borneol was the most important constituent in *Bomeolum* and had a neuroprotective effect on cerebral ischemia, but it can hardly be detected by using HPLC. Therefore, borneol was also included as a candidate component for further analyses in this study. Detailed information and structures of these components are provided in Supplementary Table S1.

### C-T Network Analysis

**FIGURE 3:**
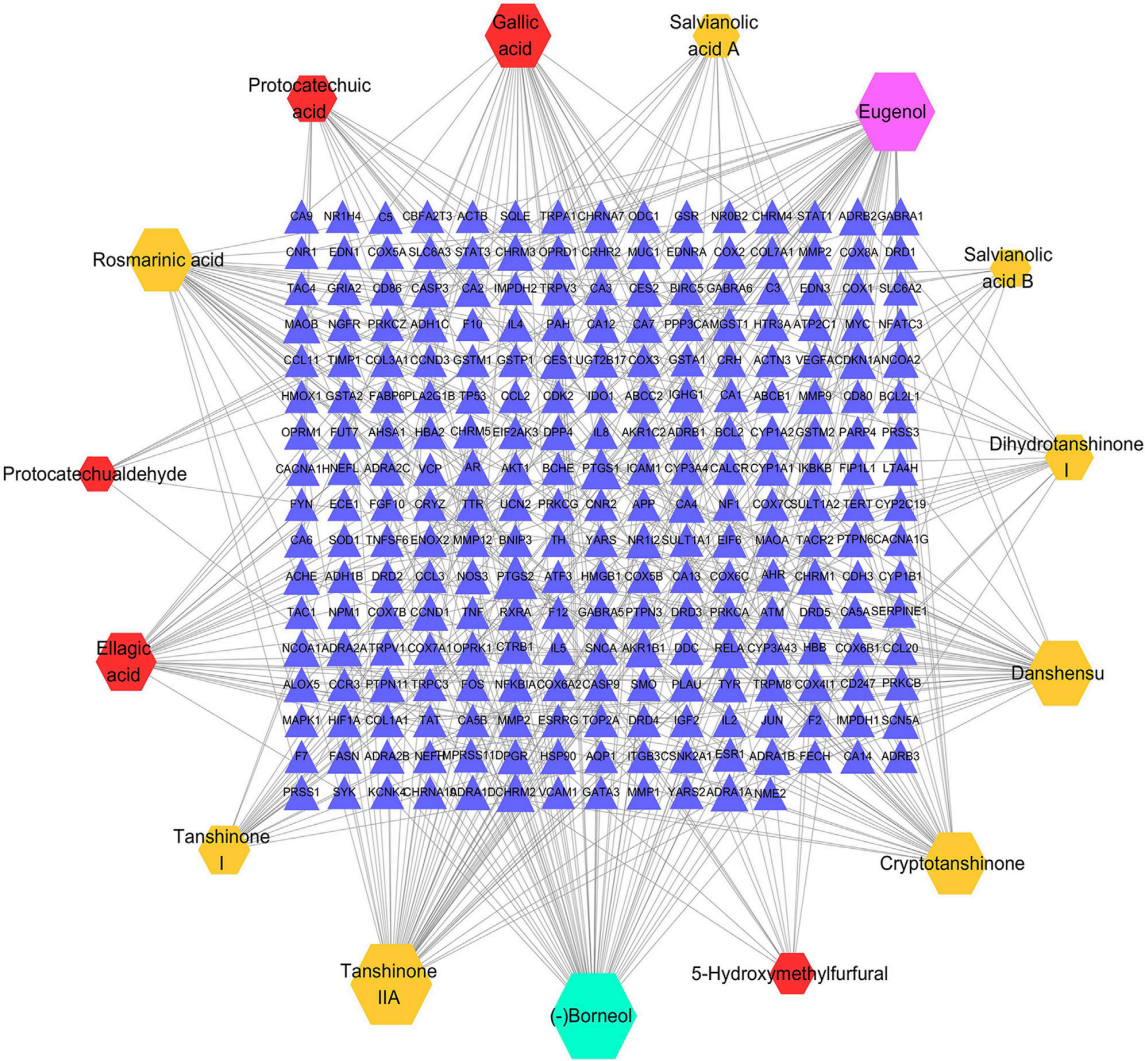
The C-T network of GXSTC, the hexagons represent the components of *Choerospondiatis fructus* (red), *Radix Salviae miltiorrhizae* (orange), *Salviae miltiorrhizae* (purple) and *Bomeolum* (blue); the blue triangles represent the targets of GXSTC components.

According to the predefined screening conditions, 252 targets related to the GXSTC components were collected (Supplementary Table S2). A visual C-T network with 267 nodes and 422 edges was established by using Cytoscape (Figure 3). The “degree” value of a node, is defined as the number of edges that connect to it in the network, representing the significance of the node in the network. Therefore, we calculated the degree value of all nodes in the C-T network, and eight components with degree values above the median value were identified, including borneol, eugenol, tanshinone IIA, danshensu, gallic acid, cryptotanshinone, rosmarinic acid, and ellagic acid.

### PPI Network Analysis

**FIGURE 4:**
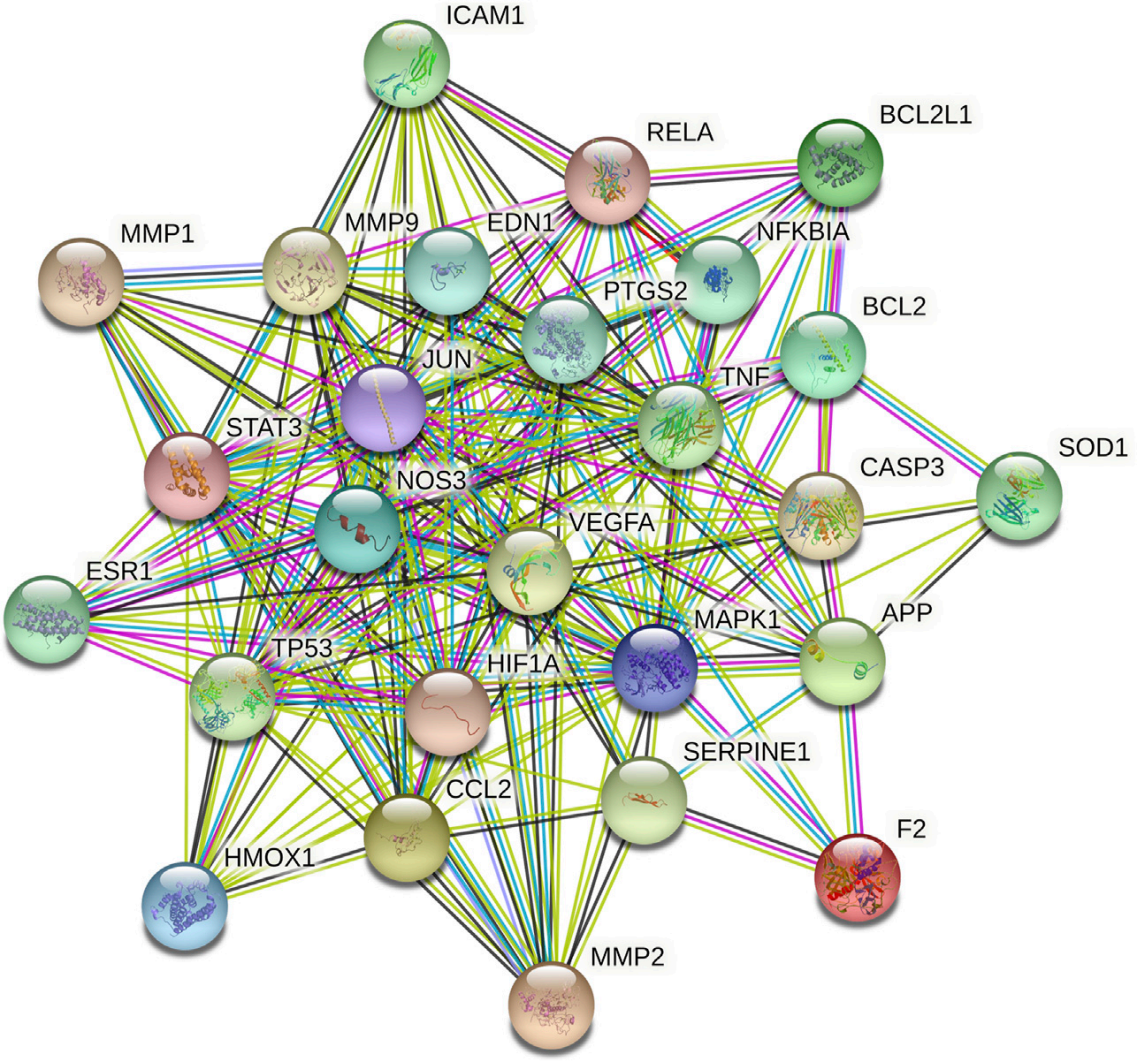
The PPI network of key targets, the different colored nodes represent different proteins; filled nodes represent some 3D structure is known or predicted; wathet and purple lines represent known protein-protein interactions; red lines represent predicted protein-protein interactions; green, black and lilac lines represent textmining, co-expression and protein homology, respectively.

A total of 462 CBVD-related targets were collected from six public databases (Supplementary Table S3). After marching the targets related to CBVDs with putative targets of GXSTC, 55 common targets were identified as candidate targets (Supplementary Table S4). Subsequently, the PPI network was constructed and visualized by using Cytoscape 3.7.2 (Figure 4). A network analyzer was performed, and the 26 genes with a degree above the median were identified as key genes in the PPI network (Supplementary Table S5). Among them, the top 10 targets with higher degree values were mitogen-activated protein kinase 1 (MAPK1), cellular tumor antigen p53 (TP53), tumor necrosis factor (TNF), beta-amyloid/A4 protein precursor (APP), transcription factor AP-1 (JUN), vascular endothelial growth factor A (VEGFA), signal transducer activator of transcription 3 (STAT3), matrix metalloproteinase-9 (MMP9), prostaglandin-endoperoxide synthase 2 (PTGS2), and endothelin-1 (EDN1).

### GO Functional and KEGG Pathway Enrichment Analysis

**FIGURE 5:**
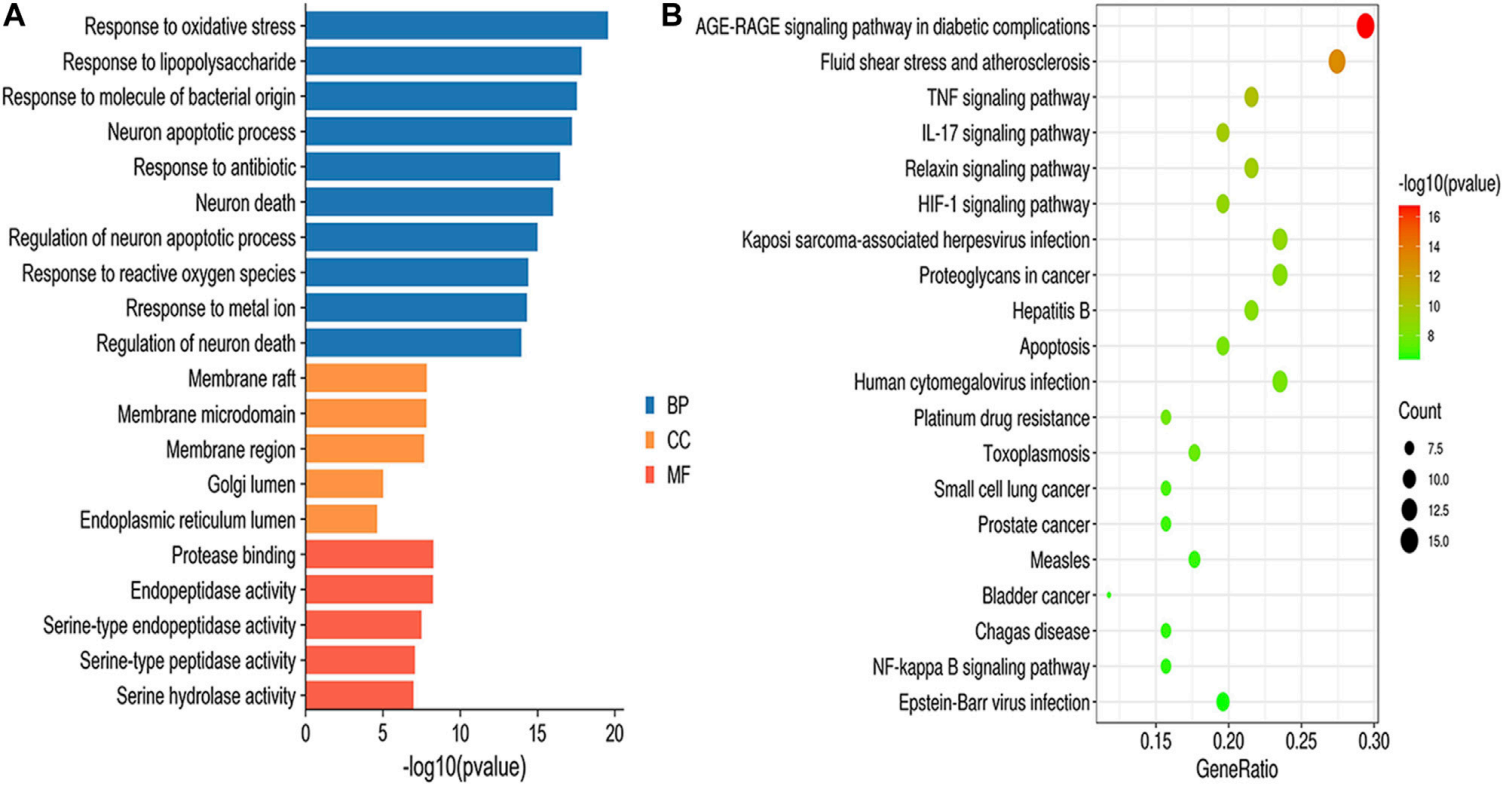
GO functional **(A)** and KEGG pathway **(B)** enrichment analysis.

GO enrichment results were divided into biological process (BP), molecular function (MF) and cellular component (CC). According to the screening criteria of *p* < 0.05, a total of 1,515 BPs, 47 CCs, 64 MFs, and 112 pathways were obtained (Supplementary Table S6), and the top 10 BPs, top 5 MFs, top 5 CCs and top 20 KEGG items are shown in Figure 5. GO enrichment analysis indicated that the BPs of GXSTC on CBVDs were related to the regulation of oxidative stress, lipopolysaccharide, and neuronal apoptotic processes. CCs were related to membrane raft, membrane microdomain, Golgi lumen, among others. MFs were involved in protease binding, serine-type endopeptidase and peptidase activity. KEGG enrichment analysis revealed that the pathways of GXSTC on CBVDs were predominantly involved in AGE-RAGE, HIF-1, TNF, IL-17 signaling pathways, fluid shear stress, atherosclerosis, and apoptosis etc.

### C-T-P Network Analysis

**FIGURE 6:**
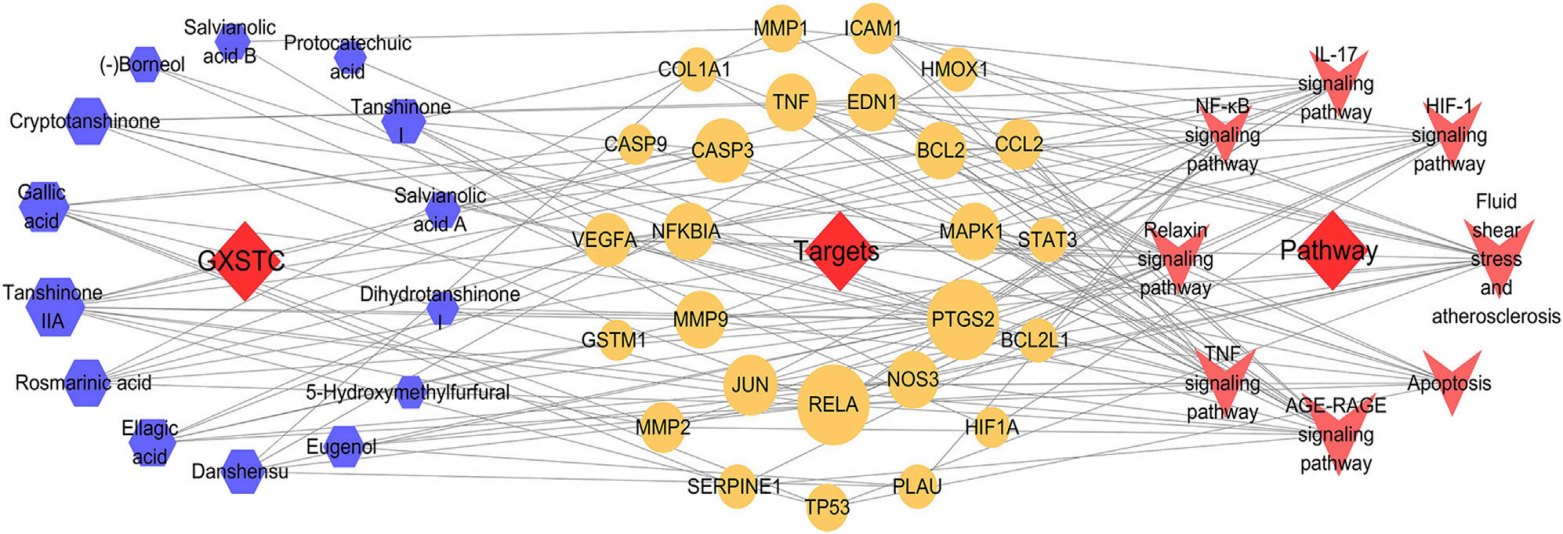
The C-T-P network of GXSTC against CBVDs. The nodes represent components (blue hexagons), targets (orange ellipses), and pathways (rose-red V shapes).

Based on the KEGG enrichment analysis results, we established a C-T-P network to systematically explain the mechanism of GXSTC on CBVDs. The network includes 48 nodes and 147 edges. The blue octagon represent 14 bioactive components of GXSTC, the orange ellipses represent 26 corresponding targets of components, and the rose-red V shapes represent the pathways related to CBVDs in the top 20 pathways (Figure 6). Seven core components with degree values above the median were identified as tanshinone IIA, cryptotanshinone, gallic acid, rosmarinic acid, ellagic acid, tanshinone I, and danshensu. Similarly, 12 core targets were identified as transcription factor p65 (RELA), PTGS2, Caspase-3 (CASP3), JUN, endothelial nitric oxide synthase (NOS3), MMP9, NF-κB inhibitor alpha (NFKBIA), TNF, EDN1, B-cell lymphoma 2 (BCL2), MAPK1, and VEGFA. These targets were selected as core targets because they play an essential role in the C-T-P network, and they are also key targets of the PPI network; therefore, these targets may be the center of the regulatory network of GXSTC against CBVDs.

### Docking Results Analysis

**FIGURE 7:**
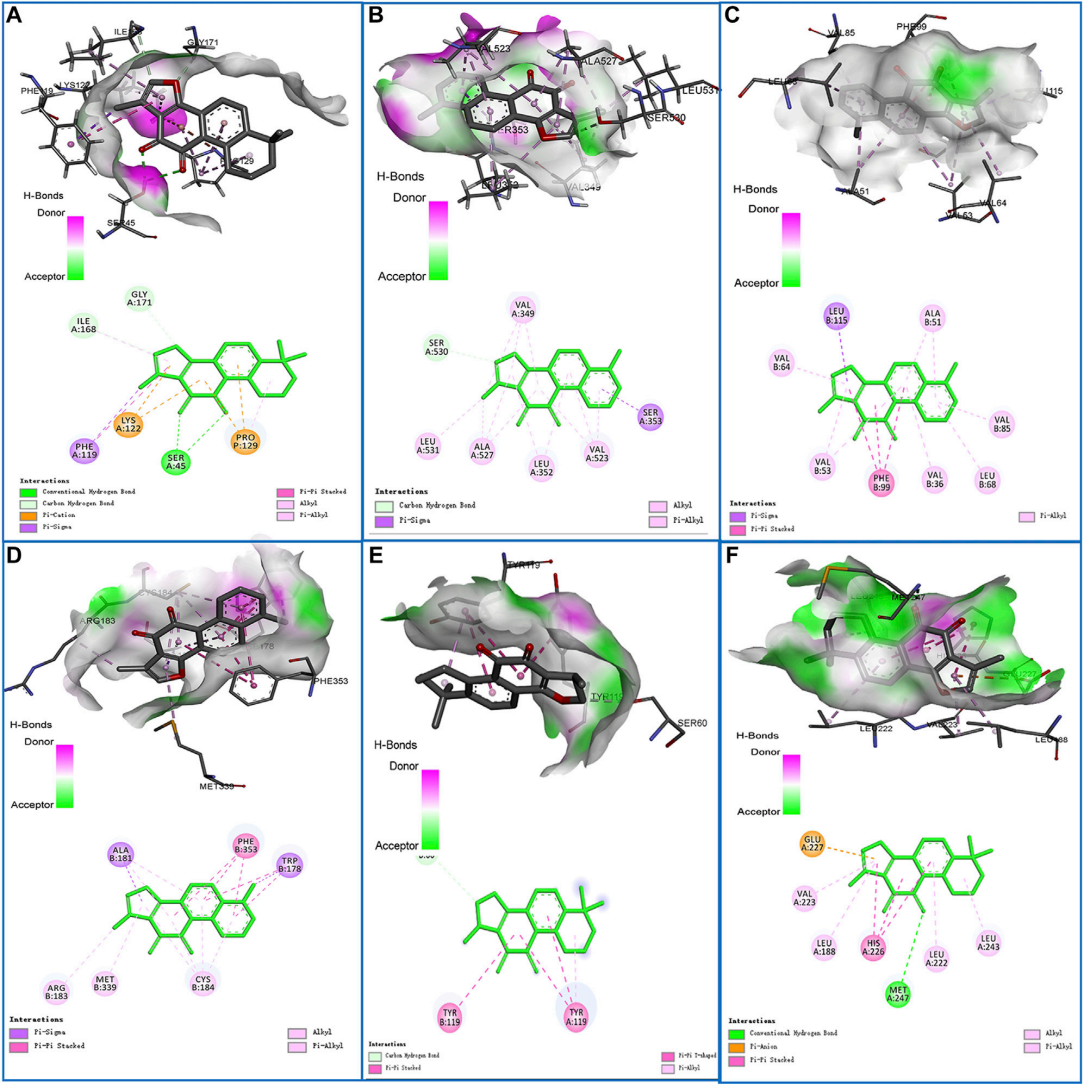
Schematic diagrams (3D and 2D) of the binding modes of representative component-targets. **(A)** Tanshinone IIA with RELA; **(B)** Tanshinone I with PTGS2; **(C)** Tanshinone I with VEGFA; **(D)** Tanshinone I with NOS3; **(E)** Tanshinone IIA with MMP9; **(F)** Cryptotanshinone with TNF.

The docking analysis between the seven core components and their corresponding core targets was carried out by using AutoDock Vina. Among them, EDN1 could not be docked because there is currently no suitable protein crystal structure available. Therefore, a total of 28 pairs of docking results were obtained, with an affinity ranging from −4.8 to −10.3 kcal/mol (Table 1). Affinity values were used to evaluate the binding interactions. The binding interaction was stronger when the docking score was lower. Usually, it is believed that an affinity < −5 kcal/mol represents a good binding activity between the component and target, and an affinity < −7 kcal/mol represents a stronger binding activity (Xia et al., 2020). In this study, there were 27 pairs of docking results satisfied the criteria of an affinity < −5 kcal/mol, and 15 pairs (15/28) met the criteria of an affinity < −7 kcal/mol. The results indicated that these core component-targets had a strong binding activity, and further confirmed the reliability of the interactions predicted by using systemic pharmacology. Among the docking results, the six pairs with the higher docking scores were selected for 3D and 2D visualization (Figures 7A–F).

**TABLE 1:** Docking scores between seven core components and their corresponding core targets.

Core target	PDB ID	GXSTC component	Affinity (kcal/mol)	Core target	PDB ID	GXSTC component	Affinity (kcal/mol)
RELA	6NV2	Original ligand	−6.4	JUN	1JNM	Tanshinone IIA	−6.1
		Tanshinone IIA	−7.3			Gallic acid	−5.4
		Cryptotanshinone	−7.1	CASP3	3H0E	Original ligand	−7.3
		Ellagic acid	−6.7			Ellagic acid	−7.3
		Rosmarinic acid	−6.6			Gallic acid	−4.8
		Danshensu	−5.3			Rosmarinic acid	−6.4
PTGS2	5KIR	Original ligand	−10.2			Tanshinone I	−7.2
		Tanshinone I	−9.3	MMP9	5CUH	Original ligand	−9.5
		Tanshinone IIA	−8.7			Tanshinone IIA	−10.3
		Cryptotanshinone	−8.3			Ellagic acid	−6.9
		Rosmarinic acid	−8.3	NFKBIA	1NFI	Tanshinone IIA	−6.4
		Danshensu	−6.8			Ellagic acid	−6.2
		Gallic acid	−6.1	TNF	2AZ5	Original ligand	−8.2
VEGFA	4QAF	Original ligand	−7.5			Cryptotanshinone	−9.2
		Tanshinone I	−10.1	BCL2	4IEH	Original ligand	−8.2
		Ellagic acid	−6.7			Tanshinone IIA	−8.0
NOS3	6PP4	Original ligand	−9.1	MAPK1	1TVO	Original ligand	−9.4
		Tanshinone I	−9.9			Rosmarinic acid	−7.9
		Ellagic acid	−7.7				

### Experimental Assessment

#### GXSTC Improves the Neurological Deficits and Reduces the Infarct Volume in MCAO-Induced Rats

**FIGURE 8:**
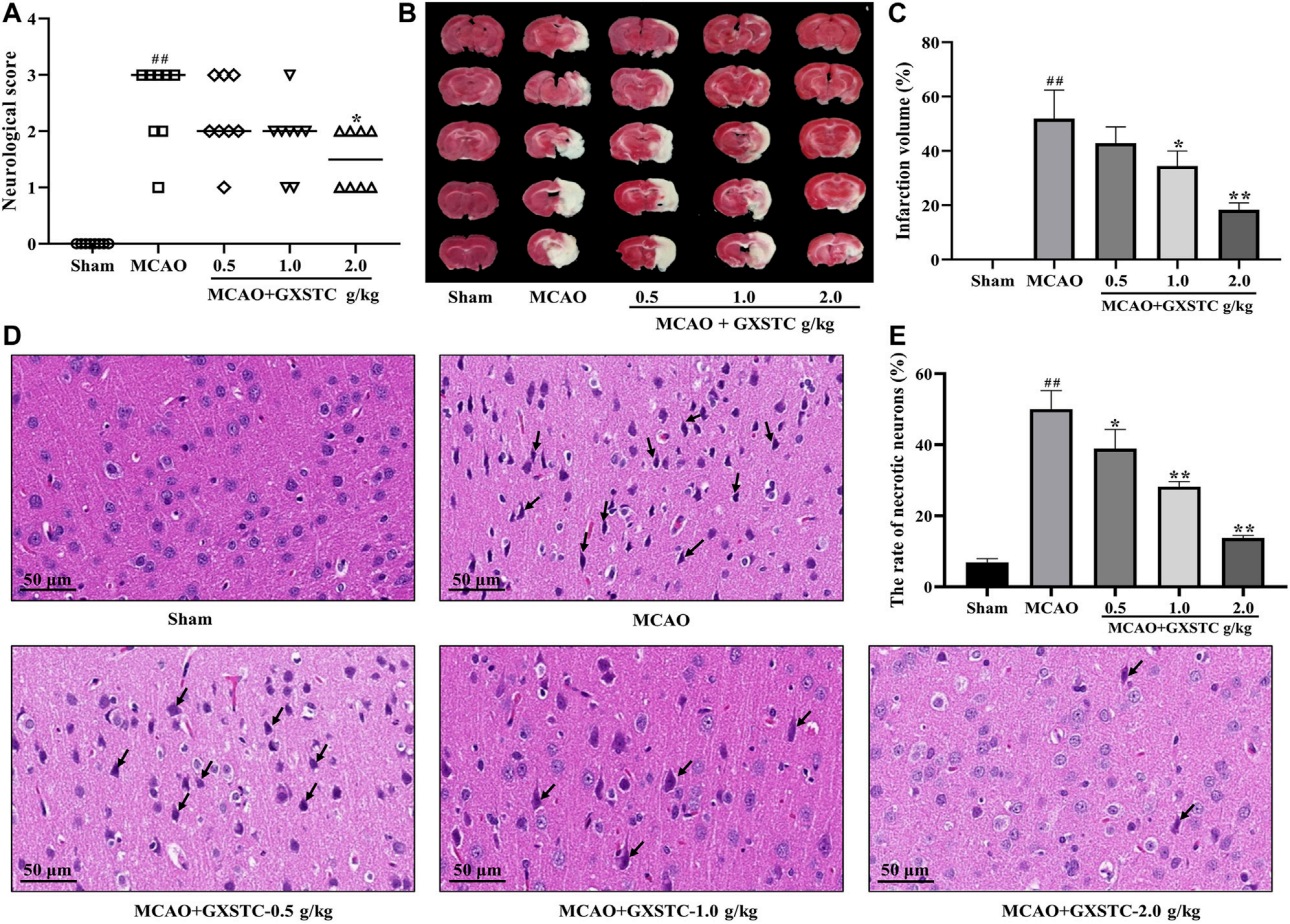
GXSTC alleviates MCAO-induced brain injury **(A)** Scatterplot of the neurological deficits in the sham, MCAO, 0.5, 1.0, 2.0 g/kg GXSTC pretreatment groups (data were expressed as median, *n* = 8) **(B)** The cerebral infarct volume of rats in each group **(C)** Statistical analysis of the rate of infarct volume in each group **(D)** H and E staining of coronal slices of the ischemic cerebral cortex, and the black arrow points to necrotic neurons **(E)** Statistical analysis results of the rate of necrotic neurons in each group. All data, except for neurologic score, were expressed as mean ± SD; ^##^ *p* < 0.01, compared with the sham group; *p < 0.05 or **p < 0.01, compared with the MCAO group.

No neurological deficits were detected in the sham group, while severe neurological deficits were observed in the MCAO group (*p* < 0.01, vs. sham group). The pretreatment with GXSTC (2.0 g/kg) significantly reduced the neurological deficits score (*p* < 0.05, vs. MCAO group; Figure 8A). Similarly, there was no detectable infarct volume in the sham group, but a large infarct volume was observed in the MCAO group (*p* < 0.01), which suggested that the MCAO model was successfully established. Furthermore, the pretreatment with GXSTC (2.0 g/kg) significantly reduced the cerebral infarct volume from 38.19 to 18.56% (*p* < 0.05, Figures 8B,C).

#### GXSTC Decreases the Rates of Necrotic Neurons After MCAO

The neuroprotective effect of GXSTC on brain injury was further confirmed by using H and E staining. As shown in Figures 8D,E, in the sham group, the neurons displayed a regular cell arrangement with a clear cell outline, and their nuclei were in the center of the cells. However, the percentage of necrotic neurons with shrunken nucleus pulposus was significantly increased in the MCAO group (*p* < 0.01, vs. sham group). After pretreatment with GXSTC at doses of 1.0 and 2.0 g/kg, the rates of necrotic neurons were markedly decreased (*p* < 0.01, vs. MCAO group).

#### GXSTC Promotes the Expression of VEGFA, e-NOS, While Inhibiting the Expression of COX-2 and c-Jun After MCAO

**FIGURE 9:**
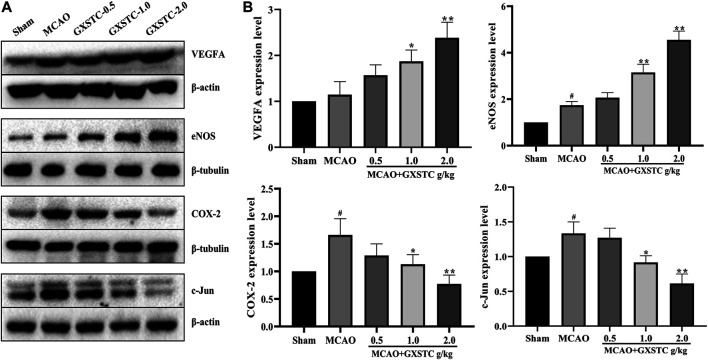
Relative expression of VEGFA, e-NOS, COX-2, and c-Jun in the cerebral cortex of the sham, MCAO, 0.5, 1.0, 2.0 g/kg GXSTC pretreatment groups were detected by using Western blot analysis. Representative bands exhibited the relative expression of VEGFA, e-NOS, COX-2 and c-JUN **(A)**; Statistical analysis results of detected proteins in each group **(B)**. Data were presented as mean ± SD. #*p* < 0.05, compared with the sham group; *p < 0.05 or **p < 0.01, compared with the MCAO group.

Because the core targets were mainly associated with inflammatory responses, regulation of vascular endothelial function, and neuronal apoptosis, we chose VEGFA, e-NOS (NOS3), COX-2 (PTGS2), and c-Jun for further investigation. As shown in Figure 9, compared with the sham group, the expression of VEGFA and e-NOS proteins was increased slightly in the MCAO group, and markedly increased in the 1.0 and 2.0 g/kg GXSTC pretreatment groups (*p* < 0.05, vs. MCAO group). With the increase in the GXSTC concentration, the expression of VEGFA and e-NOS gradually increased. Moreover, compared with the sham group, the expression of COX-2 and c-Jun was markedly increased in the MCAO group. However, the pretreatment with 1.0 and 2.0 g/kg GXSTC could significantly decreased the expression of COX-2 and c-Jun (*p* < 0.01, vs. the MCAO group).

## Discussion

GXSTC, which is derived from a Mongolian medicine formulation, has been clinically proven to be an effective and safe TCM for the treatment of CBVDs. However, the bioactive components and multi-target mechanisms of GXSTC have not yet been clearly elucidated. In this study, 15 active components in GXSTC were identified by using HPLC-DAD and a literature research. Fifty-five common targets were screened by matching the potential targets of GXSTC with the CBVD-associated targets. Twelve core targets and seven core components were identified by using PPI and C-T-P network analysis. Enrichment analysis results predicted that the therapeutic effects of GXSTC against CBVDs may be involved in the regulation of vascular endothelial function, inflammatory response and neuronal apoptosis. Finally, molecular docking and various experiments (including neurological scale scoring, TTC staining, H and E staining, and western blotting) were carried out to assess the systems pharmacology prediction.

The chemical components of TCM are characterized by complex, various types, and a constant or trace composition coexistence, but the main components of TCM are often the material basis of their efficacy. In this study, we identified 15 main components of GXSTC by using HPLC-DAD combined with a literature research, which was consistent with the results of previous studies (Zhou et al., 2016). Moreover, 13 of them had a high oral bioavailability (OB) in the TCMSP database, and their OB values are listed in Supplementary Table S1. Several studies have elucidated and identified the GXSTC components using liquid chromatography/mass spectrometry (LC/MS) *in vitro* and *in vivo* (Gao et al., 2017; Liu et al., 2018). According to their findings, the 15 main components we identified were also determined using LC/MS *in vitro*, and 12 of 15 main components were identified in rat plasma. These findings further suggested that these main components may be the key substances via which GXSTC exerts its efficacy *in vivo*.

The results of the topological analysis of the C-T-P network indicated seven core components with the higher degree values: tanshinone IIA, cryptotanshinone, gallic acid, rosmarinic acid, ellagic acid, tanshinone I, and danshensu. Several previous studies, including our own, have shown that phenolic acids, such as gallic acid (Nabavi et al., 2016), rosmarinic acid (Cui et al., 2018), and ellagic acid (Ding et al., 2014), exhibit neuroprotective effects against cerebral ischemic injury by modulating oxidative stress. In addition, our previous study indicated that danshensu has a neuroprotective effect on cerebral ischemia/reperfusion injury, and the mechanism may inhibit the apoptosis process by regulating the PI3K/Akt pathway (Guo et al., 2015). Tanshinone IIA, cryptotanshinone, and tanshinone I are major lipid-soluble components of *Radix Salviae miltiorrhizae* that can attenuate permanent cerebral ischemia by inhibiting thrombosis formation, platelet aggregation, and by activating the PLC/PKC pathway in rats (Fei et al., 2017). Taken together, these findings indicate that the core components of GXSTC are effective in treating CBVDs.

In addition, we identified 12 core targets of GXSTC on CBVDs using network analysis, and these targets were primarily related to inflammatory responses (RELA, PTGS2, NFKBIA, JUN, TNF, and MMP9), neuronal apoptosis (CASP3, JUN, BCL2, and MAPK1), and vascular endothelial function (VEGFA, NOS3, and EDN1). Inflammation is a hallmark of stroke pathology and contributes to the aggravation of brain injury (Lambertsen et al., 2012). PTGS2, also known as cyclooxygenase-2 (COX-2), is the principal isozyme responsible for the production of inflammatory prostaglandins, and its inhibition may help improve the enlargement of the infarction after CBVDs. In addition, PTGS2 can be linked to six core components of GXSTC and multiple signaling pathways; thus, it may play an essential role in GXSTC against CBVDs (Jiang et al., 2011). JUN (c-Jun), which is a subunit of the AP-1 transcription factor, plays an essential role in neuronal apoptosis and inflammation. Generally, c-Jun can be highly activated after cerebral ischemia, and the expression level of c-Jun is significantly increased in the cerebral cortex (Raivich and Behrens., 2006). MMP-9 belongs to the subfamily of MMPs that play a key role in blood-brain barrier injury and inflammatory processes after cerebral ischemia. Previous research has suggested that GXSTC can downregulate the expression of MMP-9 receptor protein in ischemic tissues (Fernández-Cadenas et al., 2012). VEGFA, a main proangiogenic factor, plays a central role in vasculogenesis and neurogenesis and is associated with neurological function in stroke recovery. When cerebral ischemia occurs, VEGFA is markedly upregulated in the ischemic penumbra area. Therefore, therapeutic angiogenesis by VEGFA is advocated as a promising treatment strategy for CBVDs (Geiseler and Morland, 2018). NOS3, also known as eNOS, plays a vital role in maintaining vascular homeostasis. When the expression of eNOS is increased, it can relax the blood vessels and prevent thrombosis (Endres et al., 2004).

GO functional and KEGG pathway enrichment analyses were performed to better understand the reciprocity of these common targets. The enriched results suggested that the molecular mechanisms of GXSTC on CBVDs were predominately involved in AGE-RAGE, HIF-1, TNF, IL-17, and the apoptosis signaling pathway. These results are consistent with those of previous studies showing that the above pathways play important roles in the progression and development of CBVDs (Wang et al., 2019). Among them, the HIF-1 signaling pathway is a major regulator of angiogenesis after CBVDs; it is observed to participate in vascular formation maintenance of vascular tension through synergistic correlations with other proangiogenic factors, such as VEGFA, eNOS, and EDN1. Therefore, the HIF-1 signaling pathway may play a crucial role in GXSTC against CBVDs (Hong et al., 2019). In addition, TNF signaling is the best-researched inflammatory pathway related to CBVDs, in which TNF-α mediates the microglial activation and triggers the activation of multiple pathways, including the NF-κB and MAPK pathways (Bradley, 2008). IL-17, which is a signature cytokine secreted by Th17 cells, is another typical pro-inflammatory cytokine involved in CBVDs, and the IL-17 pathway also plays a vital role in the inflammatory injury processes of CBVDs (Swardfager et al., 2013).

To further investigate the interactions between the core components and their corresponding core targets, molecular docking studies were performed to elucidate the binding modes. The results indicated that almost all of core components-targets had a good binding activity (affinity < −5 kcal/mol), and more than half of them had a stronger binding activity (affinity < −7 kcal/mol), which further confirmed the reliability of the systemic pharmacology prediction. More importantly, in MCAO-injured rats, GXSTC significantly improved the neurological function, reduced the infarct volume, and decreased the rate of impaired neurons in a dose-dependent manner. In addition, we selected vascular function-related proteins (VEGFA and eNOS), inflammation-related proteins (COX-2, c-Jun), neuronal apoptosis-related protein (c-Jun) from 12 core targets to further assess the molecular mechanisms. These proteins were also enriched in the HIF-1, TNF, and IL-17 signaling pathways, and may be the center of the regulatory network of GXSTC against CBVDs. Western blot analyses indicated that GXSTC markedly upregulated the expression of VEGFA and e-NOS, and downregulated the expression of COX-2 and c-Jun in MCAO-induced rats. These findings provide the evidence that GXSTC exerts a multi-target synergetic effect on CBVDs by maintaining vascular endothelial function, inhibiting neuronal apoptosis and inflammatory processes. However, identifying the core components by using network pharmacological analysis alone has some limitations, such as that it neither reflect the effect intensity against CBVDs of these core components, nor illuminate the correlation between their concentration and efficacy. Therefore, these core components are worthy of further exploration. In addition, the three doses of GXSTC (0.5, 1.0, and 2.0 g/kg per day) used in this study are comparatively high and it remains unclear whether these doses will result in artefacts in our model. They are equivalent to 80, 160, and 320 mg/kg per day in humans, respectively (converted by using the body surface area) (Administration, F. D., 2005), so a lower dose range needs to be studied in further investigation. Also, the specific neuroprotection mechanisms of GXSTC in CBVDs, as well as the targets and pathways acting with the active components still need to be further confirmed in follow-up studies.

## Conclusion

In this study, an integrated systems pharmacology approach was used to investigate the bioactive components, therapeutic targets and explore the pharmacological mechanisms of GXSTC in treating CBVDs. The results suggested that the multi-target synergetic mechanism of GXSTC in CBVDs mainly involved three therapeutic modules, including maintaining vascular endothelial function, inhibiting neuronal apoptosis and inflammatory processes. Then, we selected the rat MCAO/reperfusion model to evaluate the protective effects of GXSTC on CBVDs and chosen several core proteins to assess the predicted molecular mechanisms, confirming the reliability of our method. In conclusion, our study gives an insight into the multi-target mechanisms by systems pharmacology approach, providing a scientific foundation for GXSTC research and its clinical application in treating CBVDs.

## Data Availability

The raw data supporting the conclusion of this article will be made available by the authors, without undue reservation, to any qualified researcher.
