# Supplementary material for: Investigation of the Multi-Target Mechanism of Guanxin-Shutong Capsule in Cerebrovascular Diseases: A Systems Pharmacology and Experimental Assessment
Source: Front Pharmacol. 2021 May 13;12:650770. doi: 10.3389/fphar.2021.650770 (PMC8155632; doi:10.3389/fphar.2021.650770)
Supplement: Supplementary file 3 [file Table1.DOCX]

**Table S1 |** A list of the 15 main components of GXSTC and their corresponding structures.

| **No.** | **Mol ID** | **Molecule Name** | **OB (%)** | **BBB** | **DL** | **MF** | **Herb** |  |
| --- | --- | --- | --- | --- | --- | --- | --- | --- |
|  | MOL000513 | Gallic acid | 31.69 | -0.54 | 0.04 |  | *Choerospondiatis fructus* |  |
|  | MOL000748 | 5-Hydroxymethylfurfural | 45.07 | -0.27 | 0.02 |  | *Choerospondiatis fructus* |  |
|  | MOL000105 | Protocatechuic acid | 38.35 | 0.21 | 0.03 |  | *Choerospondiatis fructus* |  |
|  | MOL001452 | Protocatechualdehyde | 25.37 | -0.17 | 0.04 |  | *Choerospondiatis fructus* |  |
|  | MOL001002 | Ellagic acid | 43.06 | -1.41 | 0.43 |  | *Choerospondiatis fructus* |  |
|  | MOL007134 | Danshensu | 36.91 | -0.62 | 0.06 |  | *Radix Salviae miltiorrhizae* |  |
|  | MOL011865 | Rosmarinic acid | 1.38 | -1.24 | 0.35 |  | *Radix Salviae miltiorrhizae* |  |
|  | MOL007074 | Salvianolic acid B | 3.01 | -2.52 | 0.41 |  | *Radix Salviae miltiorrhizae* |  |
|  | MOL007136 | Salvianolic acid A | 2.96 | -1.62 | 0.7 |  | *Radix Salviae miltiorrhizae* |  |
|  | MOL007101 | Dihydrotanshinone I | 45.04 | 0.43 | 0.36 |  | *Radix Salviae miltiorrhizae* |  |
|  | MOL007088 | Cryptotanshinone | 52.34 | 0.51 | 0.4 |  | *Radix Salviae miltiorrhizae* |  |
|  | MOL007157 | Tanshinone I | 29.27 | 0.53 | 0.36 |  | *Radix Salviae miltiorrhizae* |  |
|  | MOL007154 | Tanshinone IIA | 49.89 | 0.7 | 0.4 |  | *Radix Salviae miltiorrhizae* |  |
|  | MOL000254 | Eugenol | 56.24 | 1.32 | 0.04 |  | *Caryophylli flos* |  |
|  | MOL000244 | (-)Borneol | 81.8 | 1.47 | 0.05 |  | *Bomeolum* |  |

* Oral bioavailability (OB), drug-likeliness (DL), blood brain barrier (BBB).
